# Value of FT3/FT4 Ratio in Prognosis of Patients With Heart Failure: A Propensity-Matched Study

**DOI:** 10.3389/fcvm.2022.859608

**Published:** 2022-04-12

**Authors:** Chuanhe Wang, Su Han, Ying Li, Fei Tong, Zhichao Li, Zhijun Sun

**Affiliations:** Department of Cardiology, Shengjing Hospital of China Medical University, Shenyang, China

**Keywords:** heart failure, long-term, mortality, prognosis, FT3/FT4 ratio, propensity-matched

## Abstract

**Aims:**

Abnormal thyroid hormone secretions can alter the manifestation and prognosis of cardiovascular disease. To assess the effect of the free triiodothyronine (FT3)/free thyroxine (FT4) ratio on the prognosis of patients with heart failure (HF), we performed a propensity-matched study on patients with well-balanced baseline characteristics.

**Methods:**

Overall, 8,887 patients with HF were divided into two groups according to the FT3/FT4 ratio. Propensity scores were calculated from each patient. A cohort comprising 2,164 pairs with high or low ratios and with 34 well-balanced baseline characteristics was then assembled. The endpoints were Cardiovascular (CV) mortality and all-cause mortality. The correlation between FT3/FT4 ratio and prognosis was assessed using matched Cox regression analyses. The mean follow-up was 3.3 years.

**Results:**

In the full pre-match cohort, 3,710 (41.7%) patients died, with 2,581 (29.0%) cases of CV mortality. In the matched-pair cohort, all-cause mortality occurred in 923 (1,238/10,000 person-years of follow-up) patients with a high ratio and 1,036 (1,484/10,000 person-years) patients with a low ratio, resulting in a matched HR of 0.841 (95% CI: 0.769–0.919; *P* < 0.001). For CV mortality, the result was 638 (856/10,000 person-years) and 714 (1,023/10,000 person-years) patients, respectively, resulting in a matched HR of 0.844 (95% CI: 0.759–0.940; *P* < 0.001). Subgroup analysis revealed that a low FT3/FT4 ratio had a greater predictive value for all-cause and CV mortality in elderly or male patients and in patients with coronary artery disease (CAD), hypertension, diabetes mellitus, HFmrEF, or HFpEF.

**Conclusions:**

A low FT3/FT4 ratio is valuable for predicting CV mortality and all-cause mortality in patients with HF.

## Introduction

Heart failure (HF) is a serious or advanced stage of any heart disease that has remained a major public health threat despite advances in medical therapy (1, 2). The prevalence of HF in developed countries ranges from 1.5 to 2.0% and increases significantly with age, with a reported prevalence of ≥10% in patients older than 70 years (3). Moreover, patients with HF have consistently been associated with a poor quality of life, reporting an in-hospital and 5-year mortality of up to 4.1% (4) and 50% (1, 2), respectively. Therefore, it is important to establish an individualized approach to improve the symptoms and prognosis of patients with HF. In particular, biomarkers can mirror the physical functions and affect patient outcomes, in addition to predicting the prognosis (5). Previous studies have even shown that biomarkers and relative mechanisms-guided management would be helpful in the prognostication, diagnosis, and treatment of patients with HF (6, 7).

Free triiodothyronine (FT3) and free thyroxine (FT4) are two major thyroid hormones that affect the physiological and pathological processes of the cardiovascular system (8–10). Studies show that low T3 syndrome has been associated with poor prognosis in patients with HF (11, 12). FT3/FT4 ratio has also been significantly correlated with adverse outcomes in patients with acute coronary syndrome (8, 9). Despite these findings, no recent literature on the effect of FT3/FT4 ratio on the prognosis of patients with HF has been found.

Traditional multivariable risk adjustment models based on regression are limited by model assumptions, which may not always be appropriate and can become a concern for residual bias and procedural transparency (13). However, propensity-matched cohort can be used to assemble two groups of patients with balanced baseline covariates (14, 15). Thus, in our present large-scale retrospective cohort study, we deduced that the FT3/FT4 ratio would be a significant biomarker for the prediction of long-term outcome in a propensity-matched cohort of patients with HF.

## Materials and Methods

### Study Population

Retrospective clinical data were collected from patients with HF hospitalized in the Department of Cardiology, Shengjing Hospital of China Medical University, Shenyang, China between 2013 and 2018. HF was diagnosed based on symptoms and signs, elevated levels of natriuretic peptides and at least one additional criterion of relevant structural heart disease (left atrial enlargement or left ventricular hypertrophy) or diastolic dysfunction (16). In accordance with the cardiac function classification published by the New York Heart Association (NYHA), heart function was divided into four levels (II–IV). Furthermore, HF with reduced ejection fraction (HFrEF) was defined as having a left ventricular ejection fraction (LVEF) of <40%, HF with mid-range LVEF (HFmrEF) was defined as having an LVEF of ≥40% but <50%, and HF with preserved LVEF (HFpEF) was defined as having an LVEF ≥50%. Patients displaying evidence of acute myocardial infarction, severe hepatic or renal failure, severe anemia, severe infection, thyroid disease (hyperthyroidism or hypothyroidism), or malignancy were excluded. This study was approved by the Shengjing Hospital of China Medical University Ethics Committee and was carried out in accordance with the principles of the Declaration of Helsinki. The ethics approval number is 2019PS594K.

### Patients

Our cohort retrospectively included 8,887 patients with HF hospitalized from January 2013 to December 2018. The investigators extracted their corresponding comprehensive clinical data from the electronic medical records. Obtained variables included patient demographics, past cardiac and non-cardiac history, physical examination results, laboratory test results, and echocardiography. All laboratory tests of the fasting peripheral venous blood samples were also taken on the day of admission or the morning after admission. LVEF was determined by echocardiography using the biplane Simpson method within 3 days of admission. In December 2020, efforts were made to determine the nature of death in each case, patients' survival status were also investigated using the population death information registration management system of the Disease Control and Prevention Center of Liaoning Province, wherein cardiac and non-cardiac death was determined in accordance with the International Classification of Diseases (ICD) code of death diagnosis. When information was not available in the system, data were obtained from the medical records, patients' physicians, or patients' relatives via telephone.

### Statistical Analysis

To avoid potential confounders and selection biases, we utilized propensity score matching. For the unmatched and matched populations, differences in the baseline characteristics were tested with chi-square and *t*-tests for categorical and continuous variables, respectively. The optimal cutoff value for the FT3/FT4 ratio was determined with the receiver operating characteristic (ROC) curve. The propensity score (PS) for FT3/FT4 category was separately calculated by a logistic regression model to reduce the selection bias. The clinically relevant variables, which had significant difference between high FT3/FT4 ratio and low FT3/FT4 ratio groups at baseline were included as covariates (Table 1). High FT3/FT4 ratio and low FT3/FT4 ratio were then matched 1:1 using the nearest neighbor method with a caliper of <0.01 without any replacement. The ability of the matching to balance baseline characteristics in high vs. low ratios was assessed using absolute standard differences and a quartile, reporting a non-significant value of <10%. The absolute standardized differences before and after matching were shown as Love plots. Primary outcomes of this study included 8-year all-cause mortality and 8-year CV mortality. Cox proportional hazards models were also used in overall cohort adjustment and matched population to estimate the association between the FT3/FT4 ratio and outcomes. Results were presented with their hazard ratio (HR) and corresponding 95% confidence interval (CI), and survival estimates were visualized using the Kaplan–Meier method. Interaction analyses were further conducted with consideration to the age, gender, CAD, hypertension, diabetes mellitus, stroke, atrial fibrillation, previous myocardial infarction, and LVEF in the matching population. All statistical analyses were performed using the R software version 3.6.1, and a two-tailed *P*-value of <0.05 was considered to be statistically significant.

**Table 1:** Baseline characteristics of patients in the high and low FT3/FT4 ratio groups before and after propensity matching.

	**Unmatched**			**Propensity-Matched**		
	**High FT3/FT4**	**Low FT3/FT4**	***P-*** **value**	**High FT3/FT4**	**Low FT3/FT4**	***P-*** **value**
*N*	5,245	3,642		2,164	2,164	
Age (years)	67.38 ± 13.27	71.05 ± 13.33	<0.001	70.40 ± 12.73	70.22 ± 13.41	0.637
Male sex, *n* (%)	2,979 (56.8%)	1,801 (49.5%)	<0.001	1,107 (51.2%)	1,110 (51.3%)	0.927
NYHA Fc			<0.001			0.426
NYHA class II, *n* (%)	1,583 (30.2%)	405 (11.1%)		339 (15.7%)	350 (16.2%)	
NYHA class III, *n* (%)	2,300 (43.9%)	1,477 (40.6%)		953 (44.0%)	984 (45.5%)	
NYHA class IV, *n* (%)	1,362 (26.0%)	1,760 (48.3%)		872 (40.3%)	830 (38.4%)	
CAD, *n* (%)	3,498 (66.7%)	2,413 (66.3%)	0.668	1,474 (68.1%)	1,457 (67.3%)	0.580
Hypertension, *n* (%)	3,317 (63.2%)	2,180 (59.9%)	0.001	1,373 (63.4%)	1,356 (62.7%)	0.592
Diabetes mellitus, *n* (%)	1,664 (31.7%)	1,301 (35.7%)	<0.001	770 (35.6%)	757 (35.0%)	0.679
Stroke, *n* (%)	914 (17.4%)	741 (20.3%)	0.001	419 (19.4%)	424 (19.6%)	0.848
Atrial fibrillation, *n* (%)	1,818 (34.7%)	1,092 (30.0%)	<0.001	678 (31.3%)	690 (31.9%)	0.695
Previous MI, *n* (%)	967 (18.4%)	704 (19.3%)	0.289	435 (20.1%)	416 (19.2%)	0.467
Current smoker, *n* (%)	1,529 (29.2%)	936 (25.7%)	<0.001	584 (27.0%)	576 (26.6%)	0.784
Alcohol-related, *n* (%)	1,014 (19.3%)	529 (14.5%)	<0.001	334 (15.4%)	355 (16.4%)	0.383
SBP (mmHg)	137.12 ± 23.64	133.37 ± 25.46	<0.001	135.74 ± 22.85	135.62 ± 23.38	0.869
DBP (mmHg)	81.97 ± 14.50	78.95 ± 14.81	<0.001	80.44 ± 13.59	80.26 ± 13.95	0.682
Heart rate (bpm)	84.98 ± 22.21	89.76 ± 25.01	<0.001	88.05 ± 21.38	87.64 ± 23.05	0.548
NT-proBNP (ng/L)	2008.00 (785.30–4300.75)	5317.00 (2374.32–10724.50)	<0.001	3414.00 (1559.00–6917.00)	3569.50 (1697.00–7296.00)	0.113
Troponin I (ug/L)	0.02 (0.01–0.06)	0.05 (0.02–0.23)	<0.001	0.03 (0.01–0.11)	0.04 (0.01–0.14)	0.057
Hemoglobin (g/L)	131.93 ± 20.72	122.04 ± 24.37	<0.001	125.96 ± 21.74	126.03 ± 22.04	0.898
BUN (mmol/L)	7.57 ± 4.02	10.30 ± 6.25	<0.001	8.67 ± 4.97	8.79 ± 4.71	0.413
Creatinine (μmol/L)	78.60 (65.00–98.00)	91.60 (72.00–121.28)	<0.001	83.00 (67.20–102.90)	84.60 (70.00–110.20)	0.064
Uric acid (μmol/L)	433.10 (336.00–468.00)	441.36 (359.98–557.50)	<0.001	441.36 (356.00–501.40)	441.36 (346.00–506.43)	0.398
Prealbumin (g/L)	0.21 ± 0.06	0.17 ± 0.06	<0.001	0.19 ± 0.05	0.19 ± 0.06	0.484
Platelets (10^∧^9L)	192.19 ± 62.94	193.70 ± 77.99	0.346	191.95 ± 70.29	189.92 ± 66.72	0.328
Albumin (g/L)	38.43 ± 4.09	35.55 ± 4.35	<0.001	36.87 ± 3.88	36.85 ± 3.92	0.842
TC (mmol/L)	4.23 ± 1.09	3.91 ± 1.14	<0.001	4.06 ± 0.70	4.06 ± 0.80	0.948
Triglycerides (mmol/L)	1.45 ± 1.04	1.16 ± 0.86	<0.001	1.26 ± 0.74	1.24 ± 0.95	0.544
HDL (mmol/L)	1.01 ± 0.30	0.93 ± 0.32	<0.001	0.96 ± 0.29	0.97 ± 0.31	0.267
LDL-C (mmol/L)	2.66 ± 0.94	2.47 ± 0.96	<0.001	2.56 ± 0.91	2.55 ± 0.96	0.828
HbA1c%	6.45 ± 1.30	6.59 ± 1.47	<0.001	6.58 ± 1.30	6.55 ± 1.37	0.441
Potassium (mmol/L)	4.06 ± 0.49	4.11 ± 0.61	<0.001	4.09 ± 0.54	4.08 ± 0.56	0.472
Sodium (mmol/L)	139.60 ± 3.20	137.89 ± 4.42	<0.001	138.92 ± 3.64	138.89 ± 3.64	0.833
Chloride ion (mmol/L)	105.73 ± 3.87	103.58 ± 5.10	<0.001	104.78 ± 4.30	104.83 ± 4.25	0.753
LVEDV (ml)	157.69 ± 62.40	159.66 ± 61.03	0.138	158.56 ± 64.27	157.38 ± 60.91	0.524
LVESV (ml)	82.71 ± 50.55	89.18 ± 49.56	<0.001	86.52 ± 50.06	85.40 ± 48.89	0.455
LVEF (%)	50.37 ± 12.12	46.70 ± 12.13	<0.001	47.99 ± 12.05	48.29 ± 11.86	0.403

*NYHA, New York Heart Association (NYHA); CAD, Coronary artery disease; Previous MI, Previous myocardial infarction; SBP, Systolic blood pressure; DBP, Diastolic blood pressure; NT-proBNP, N-terminal pro-B-type natriuretic peptide; BUN: Blood urea nitrogen; TC, Total cholesterol; HDL, High density lipoprotein; LDL-C, Low density lipoprotein cholesterol; LVEDV, Left ventricular end-diastolic volume; LVESV, Left ventricular end-systolic volume; LVEF, Left ventricular ejection fraction*.

## Results

### Baseline Characteristics

**Figure 1:**
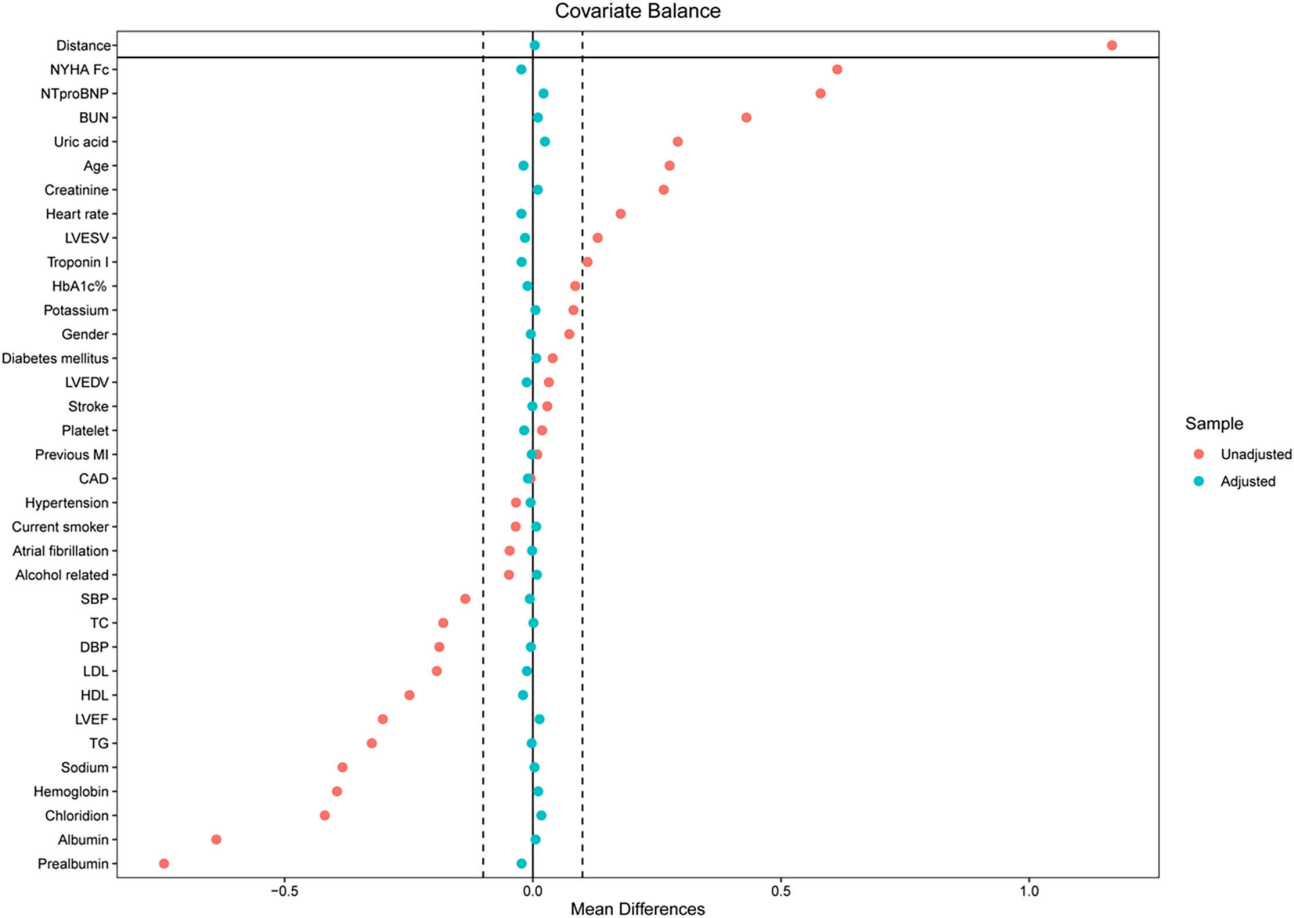
Love plots for absolute standardized differences for baseline covariates of patients between high and low FT3/FT4 ratio group, before and after propensity score matching.

A total of 8,887 patients met the inclusion criteria. Patients in the cohort had a mean age of 69 (± 13) years, and 53.8% were male. The median follow-up time was 3.3 years (2–8 years).The optimal cutoff value for FT3/FT4 is 0.233, which was determined with the receiver operating characteristic (ROC) curve, with a area under the ROC curve (AUC) of 0.668. According to this cutoff value, FT3/FT4 is divided into two groups: the high FT3/FT4 (0.233~0.563) and low FT3/FT4 ratio group (0.085~0.233). The propensity score for the FT3/FT4 category was separately calculated using a logistic regression model, and the clinically relevant variables listed in Table 1 were used as covariates. Prior to matching, patients in the high FT3/FT4 ratio group were found to be younger, more likely to be male, had a more severe NYHA heart function, and had lower NT-proBNP levels than those in the low FT3/FT4 ratio group (Table 1, Figure 1). Other significant imbalances in baseline characteristics before matching and the balances after matching are displayed in Table 1, Figure 1. After matching, absolute standardized differences for all measured covariates were <10% (usually 5%), indicating significant covariate balance between the two groups (Figure 1).

### FT3/FT4 Ratio and Mortality

Overall, in the full pre-match cohort of 8,887 patients, 3,710 (41.7%) patients died, with 2,581 (29.0%) cases of CV mortality, during a median follow-up of 3.2 years. All-cause mortality occurred in 1,655 (rate, 850/10,000 person-years of follow-up) patients with a high FT3/FT4 ratio and 2,055 (rate, 1,981/10,000 person-years) patients with a low ratio, resulting in an unadjusted HR of 0.442 (95% CI: 0.414–0.471; *P* < 0.001) and an adjusted HR of 0.810 (95% CI: 0.752–0.872; *P* < 0.001) (Table 2). Meanwhile, CV mortality occurred in 1,117 (rate, 573/10,000 person-years) patients with a high FT3/FT4 ratio and 1,464 (rate, 1,412/10,000 person-years) patients with a low ratio, resulting in an unadjusted HR of 0.422 (95% CI:0.391–0.457; *P* < 0.001) and an adjusted HR of 0.795 (95% CI: 0.727–0.870; *P* < 0.001) (Table 2).

**Table 2:** Hazard ratio for FT3/FT4 ratio and all-cause/CV mortality.

	**Rate per 10,000 person-years** **(events/total follow-up years)**		**Absolute rate difference** **(per 10,000 person-years)**	**Hazard ratio** **(95% CI)**	***P-*** **value**
	**High FT3/FT4**	**Low FT3/FT4**			
*Before matching*	*n* = 5,245	*n* = 3,642			
**All-cause mortality**					
Unadjusted	850 (1,655/19,481)	1,981 (2,055/10,371)	−1,131	0.442 (0.414–0.471)	<0.001
Adjusted				0.810 (0.752–0.872)	<0.001
**CV mortality**					
Unadjusted	573 (1,117/19,481)	1,412 (1,464/10,371)	−839	0.422 (0.391–0.457)	<0.001
Adjusted				0.795 (0.727–0.870)	<0.001
*After matching*	*n* = 2,164	*n* = 2,164			
All-cause mortality	1,238 (923/7,453)	1,484 (1,036/6,980)	−246	0.841 (0.769–0.919)	<0.001
CV mortality	856 (638/7,453)	1,023 (714/6,980)	−167	0.844 (0.759–0.940)	0.002

*CV, cardiovascular disease*.

**Figure 2:**
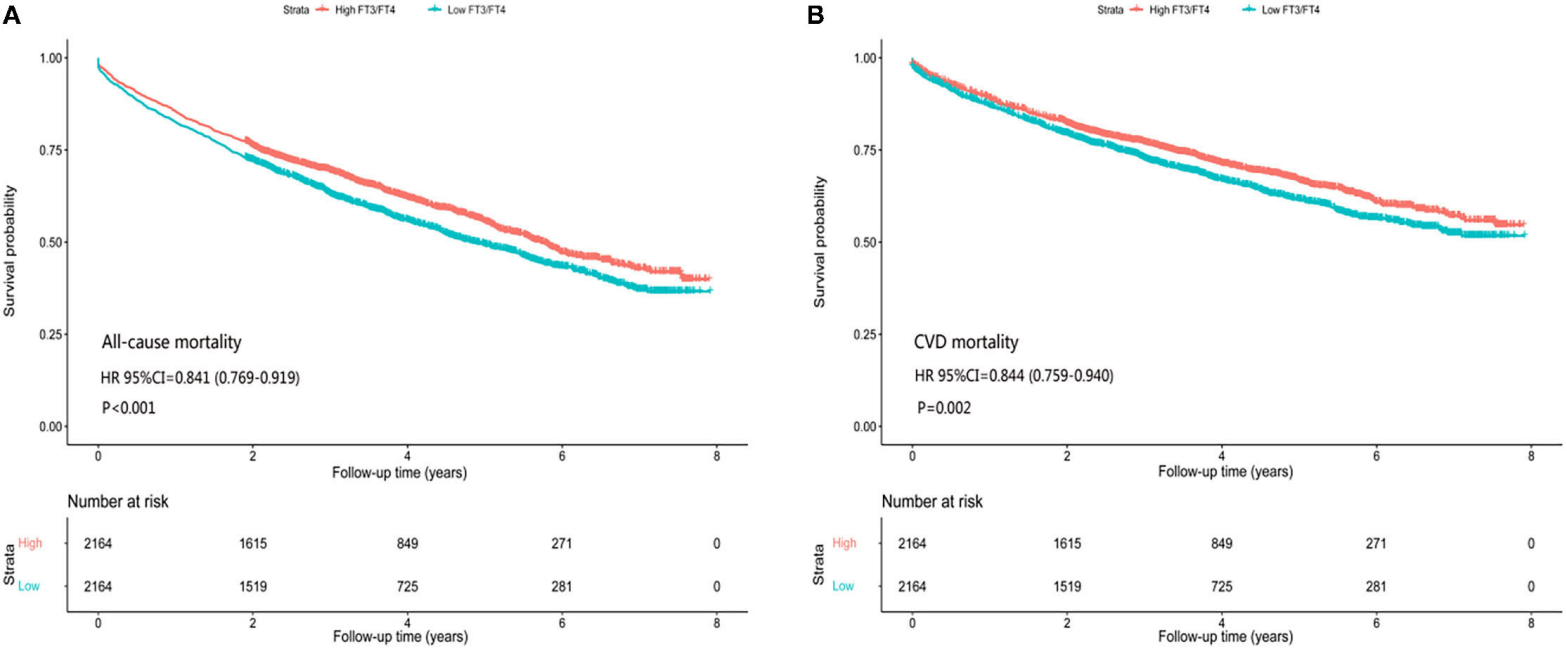
Association of high and low FT3/FT4 ratio with all-cause/CV mortality in subgroups of propensity score-matched patients, **(A)** all-cause mortality, **(B)** CV mortality.

In the matched-pair cohort of 2,164 patients, all-cause mortality occurred in 923 (rate, 1,238/10,000 person-years) patients with a high FT3/FT4 ratio and 1,036 (rate, 1,484/10,000 person-years) patients with a low ratio, resulting in a matched HR of 0.841 (95% CI: 0.769–0.919; *P* < 0.001) (Figure 2A, Table 2). For CV mortality, this was observed in 638 (rate, 856/10,000 person-years) patients with a high FT3/FT4 ratio and 714 (rate, 1,023/10,000 person-years) patients with a low ratio, resulting in a matched HR of 0.844 (95% CI: 0.759–0.940; *P* < 0.001) (Figure 2B, Table 2).

Findings from our sensitivity analysis indicate that the covariate (FT3/FT4), which is a predictor of mortality in patients with HF, may potentially explain the association between HF and mortality.

**Figure 3:**
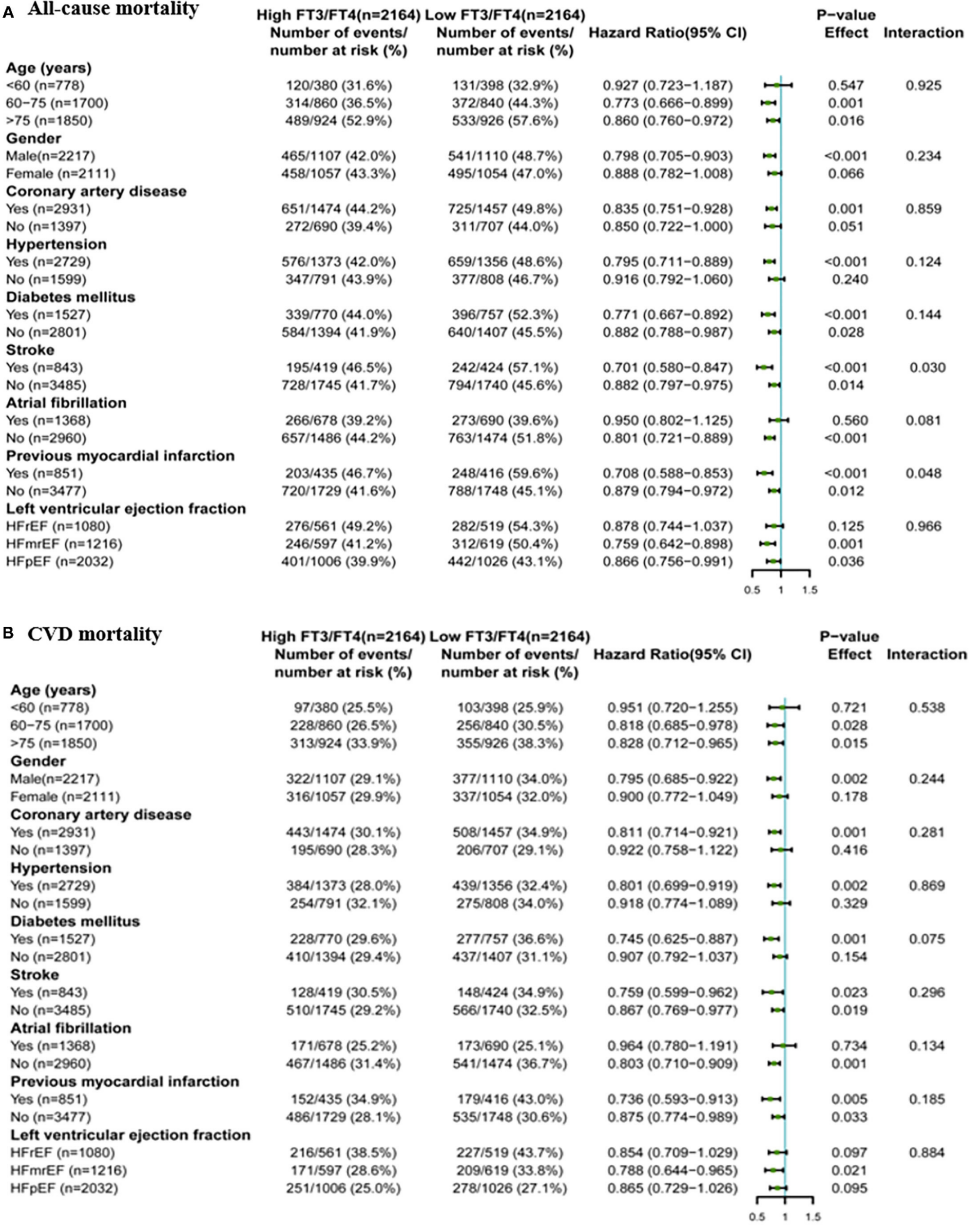
Association of high and low FT3/FT4 ratio with all-cause/CV mortality in subgroups of propensity score-matched patients, **(A)** all-cause mortality, **(B)** CV mortality.

Correlations between the FT3/FT4 ratio and all-cause/CV mortality among various subgroups are displayed in Figure 3. Generally, a low FT3/FT4 ratio had a greater predictive value for all-cause mortality and CV mortality in elderly or male patients and in patients with CAD, hypertension, diabetes mellitus, HFmrEF, or HFpEF.

## Discussion

In our retrospective cohort of 8,887 patients, 3,710 (41.7%) patients died, with 2,581 (29.0%) cases of CV mortality, during a median follow-up of 3.2 years. In the matched-pair cohort of 2,164 pairs of patients with high and low FT3/FT4 ratios and with 34 balanced baseline characteristics, we found that all-cause and CV mortality in patients with HF could be predicted independently using a low FT3/FT4 ratio. Specifically, the hazard ratio of long-term all-cause mortality for patients with a high FT3/FT4 ratio was 0.841 times less than that in patients with a low FT3/FT4 ratio. This was similarly seen in CV mortality, with a value of 0.844.

Baseline analysis showed that a lower FT3/FT4 ratio was associated with worse heart function and clinical characteristics, including advanced age; higher rates of diabetes and stroke; increased troponin I and NT-probNP; and decreased hemoglobin, albumin, and sodium; all of which may be related to poor prognosis in HF. Numerous studies have also shown that reduced FT3 was associated with increased cardiovascular morbidity and mortality (including HF) (10, 12), and high FT4 level within normal thyroid function has been correlated with HF and sudden cardiac death (17, 18). In particular, Kannan et al. demonstrated that higher FT4 and lower total triiodothyronine were associated with an increased risk of the composite end point for left ventricular assist device implantation, heart transplantation, or all-cause mortality (19). Furthermore, previous studies have indicated that a decreased FT3/FT4 ratio is associated with an increased risk of long-term all-cause mortality, cardiovascular mortality, and major adverse cardiovascular events in patients with CAD (9, 20).

The cardiovascular system is an important target organ of thyroid hormones, as their main effect on this system increases the heart rate, enhances myocardial contractility, reduces circulation resistance, and relieves cardiac afterload (21, 22). Thyroid hormone abnormalities, such as thyroid function hyperfunction, hypothyroidism, and low T3 syndrome, can influence the clinical manifestations and outcomes of cardiovascular disease (11). Subclinical hyperthyroidism, subclinical hypothyroidism, and low T3 syndrome are associated with high risk of atrial fibrillation and increased mortality of patients with cardiac disease (23, 24). Even a minor alteration in thyroid hormone concentration will affect the pathological and physiological process of the cardiovascular system (25, 26). Among these thyroid hormones, FT3 and FT4 are usually more sensitive and clinically relevant than T3 and T4, given that former are the physiologically active forms of the latter (27).

Previous studies have demonstrated that the FT3/FT4 ratio could reflect deiodinase activity (28), so the decline of this ratio may reflect the reduced transformation from T4 to T3 in the peripheral blood (27, 29). Therefore, the possible mechanisms of the influence of FT3/FT4 ratio on HF prognosis can be as follows. First, insufficient FT3 transformation leads to more obvious oxidative stress damage of the endoplasmic reticulum and impaired utilization of ATP by cardiomyocytes, resulting in an impaired systolic function and development of HF (30). Second, there are specific T3 receptors in the myocardium, and the decrease of FT3 may lower myocardial contractility and increase susceptibility to arrhythmia, possibly resulting in death in patients with HF (12). Third, low levels of FT3 are related to the increase of right atrial pressure, pulmonary artery pressure, and pulmonary capillary wedge pressure, as well as the decrease of ejection fraction and cardiac index, which results in myocardial fibrosis, ventricular remodeling, and myocardial perfusion and metabolism abnormalities (30). Furthermore, the FT3/FT4 ratio may be an important predictor of metabolic disease (31) and acute myocardial infarction (20).

Given the effect of thyroid hormones on the prognosis of heart failure, some scholars believe that replacement therapy, regulation of deiodinase activity, and heart-specific thyroid receptor agonists are potential treatments for HF (32). However, the potential benefits of thyroid hormone supplementation for these patients should be weighed against the risks of overtreatment. Moreover, the tolerability and safety of the aforementioned treatment regimens need further studies to confirm their viability.

Despite the large sample of the present study, a few limitations were noted. First, although we eliminated the influence of confounding factors and selection bias using propensity-matched scoring, we consequently missed a large portion of real-world data. Second, FT3 and FT4 were measured at baseline without dynamic monitoring, which may have limited the accuracy of the results. Lastly, we failed to mention and analyze the drug use of the included patients. This is important since iodized contrast agents, including amiodarone and glucocorticoids, might have affected thyroid function.

In conclusion, this propensity-matched study revealed that a low FT3/FT4 ratio had a greater predictive value for all-cause mortality and CV mortality, especially in elderly and male patients and in patients with CAD, hypertension, diabetes mellitus, HFmrEF, or HFpEF.

## Data Availability Statement

The original contributions presented in the study are included in the article/supplementary material, further inquiries can be directed to the corresponding author/s.

## Ethics Statement

The studies involving human participants were reviewed and approved by Shengjing Hospital of China Medical University Ethics Committee and was carried out in accordance with the principles of the Declaration of Helsinki. The ethics approval number is 2019PS594K. Written informed consent for participation was not required for this study in accordance with the national legislation and the institutional requirements.

## Author Contributions

CW and ZS conceived and designed the study and wrote the paper. CW, SH, and YL extracted and sorted clinical data. ZL and FT analyzed the data. All authors contributed to the article and approved the submitted version.

## Funding

The present study was financially supported by a grant from the Science and Technology Program of Liaoning Province (No. 2018225003).

## Conflict of Interest

The authors declare that the research was conducted in the absence of any commercial or financial relationships that could be construed as a potential conflict of interest.

## Publisher's Note

All claims expressed in this article are solely those of the authors and do not necessarily represent those of their affiliated organizations, or those of the publisher, the editors and the reviewers. Any product that may be evaluated in this article, or claim that may be made by its manufacturer, is not guaranteed or endorsed by the publisher.
